# Smart and Sustainable Regeneration of Fouled Desalination Membranes Using Artificial Intelligence

**DOI:** 10.1002/gch2.202500235

**Published:** 2025-07-14

**Authors:** Muhammad Mubashir, Mustakeem Mustakeem, Ammar Alnumani, Abdulrahman Abutaleb, Ali Hamoud Naji Sumayli, Tausif Ahmad, Muhammad Rizwan Azhar

**Affiliations:** ^1^ Water Technologies Innovation Institute & Research Advancement Saudi Water Authority WTIIRA‐SWA Jubail 35417 Saudi Arabia; ^2^ Department of Chemical & Biological Engineering American University of Sharjah Sharjah 26666 UAE; ^3^ School of Engineering Edith Cowan University 270 Joondalup Drive Joondalup Western Australia 6027 Australia

**Keywords:** artificial intelligence (AI), desalination, membrane regeneration, membranes

## Abstract

During the desalination process, scaling, fouling, and degradation are associated issues that lead to a drop in the separation performance of membranes. Membrane regeneration emerges as a critical technology in which upcycling and downcycling can offer a promising avenue for promoting sustainable membrane lifecycle management. Multiple research papers and reviews have critically analyzed the regeneration of membranes, which explains the end‐of‐cycle assessment and cost analysis of membrane recycling. However, challenges associated with the conventional and innovative regeneration processes are not yet analyzed. The potential impact of artificial intelligence (AI) on membrane regeneration is not explained in the literature. This review paper aims to explore the synergistic relationship between AI and membrane regeneration, elucidating the principles, challenges, opportunities, and emerging trends in this rapidly evolving field. By examining the role of AI techniques in enhancing the understanding, monitoring, and control of regeneration membrane processes, as well as their applications in optimizing regeneration strategies and addressing end‐of‐life considerations, this paper seeks to provide insights into the transformative potential of AI in reshaping the landscape of membrane regeneration.

## Graphical abstract

Artificial intelligence (AI) is transforming membrane regeneration by addressing challenges like fouling, aging, and scalability. This study compares conventional and AI‐driven approaches, highlighting advancements through models like CNN, RNN, and IoT integration. While AI offers promising improvements in predictive maintenance and optimization, limitations in data quality, interpretability, and generalization remain key hurdles.

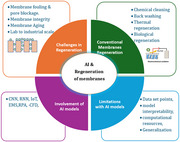

## 1 Introduction

**Figure 1:**
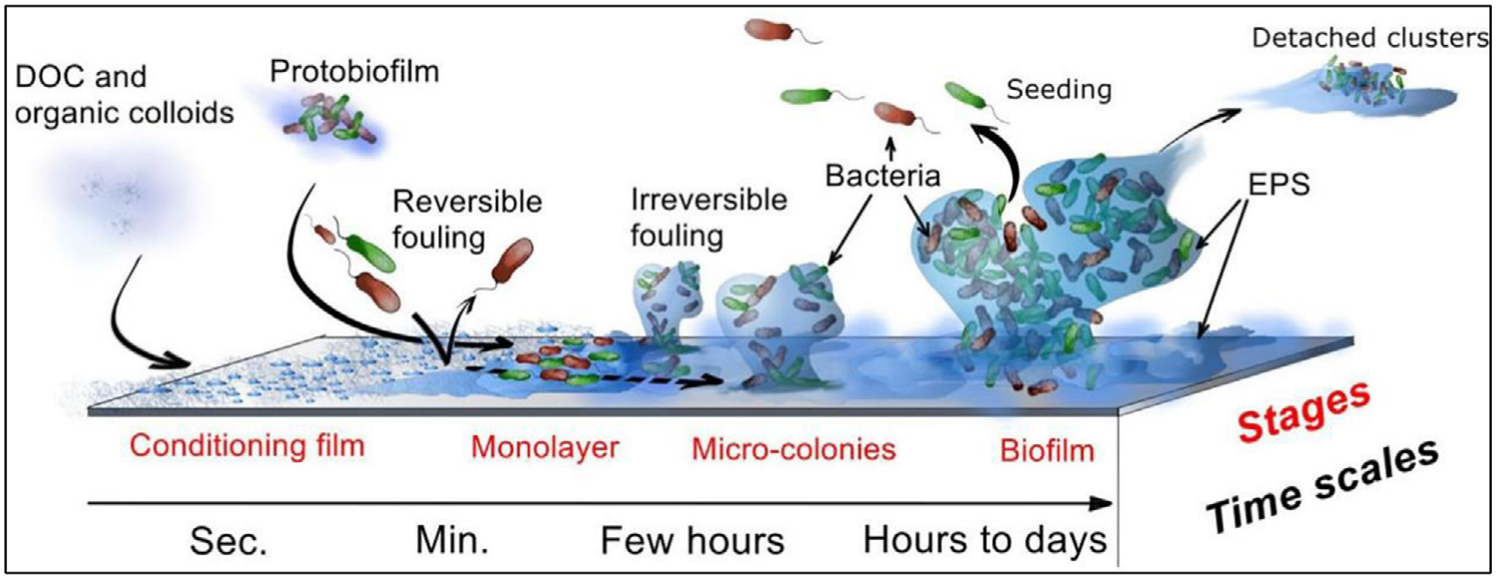
Formation of biofilm on membrane surface, reproduced with permission from ref. [11].

Membrane technology is regarded as the keystone for the desalination market as it offers low cost and environmentally friendly solutions.^[^ ^1^, ^2^, ^3^ ^]^ Polymeric membranes are effective and have been widely used at the industrial level due to low cost, easy maintenance and ease of scale‐up.^[^ ^4^, ^5^ ^]^ However, fouling, degradation, scaling, and low efficiency of membranes have emerged as challenges for the membrane systems, leading to increased operational expenses.^[^ ^2^, ^6^ ^]^ Challenges associated with the termination of membrane life are membrane disposal, recycling of membrane, incineration, and environmental impact.^[^ ^7^ ^]^ The cost involved in the membrane regeneration could reduce the operational costs and environmental impacts. Furthermore, advancement in membrane regeneration can open up new possibilities for sustainable membrane lifecycle.^[^ ^8^ ^]^ In the past few years, integration of artificial intelligence (AI) and membrane processes has been identified as an exciting area to transform the membrane rejuvenation, membrane replacement, and membrane performance enhancement.^[^ ^9^ ^]^ Membrane regeneration appears as another key which addresses the problem of recovery of membrane performance and its characteristics when it get fouled and degraded during the desalination process.^[^ ^10^ ^]^ Bio‐ film formation on the surface of the membrane is shown in **Figure** **1**.^[^ ^11^ ^]^ Recently, different membrane regeneration techniques including chemical cleaning, physical cleaning, biological regeneration, and thermal regeneration have been used.^[^ ^12^, ^13^ ^]^ Regeneration methods often rely on empirical rules, trial‐and‐error approaches, and manual interventions, which may be time‐consuming, inefficient, and suboptimal.^[^ ^13^ ^]^ Therefore, novel AI technologies have been adopted to optimize the regeneration process of membranes. AI‐driven regeneration techniques offer a data‐driven, adaptive, and systematic approach to identify, diagnose, and mitigate regeneration issues. In addition, it optimized the cleaning protocols and enhance regeneration efficiency in short period of time.^[^ ^1^, ^14^ ^]^ AI integrated with remote monitoring platforms, internet of things (IoT) devices, and sensor technologies could enable real‐time control and facilitate collaborative learning across multiple membrane regeneration systems.^[^ ^1^ ^]^ However, integration of AI models with membrane regeneration process is challenging and requires significant effort.

Various review papers^[^ ^15^, ^16^, ^17^ ^]^ have recently summarized the cleaning of membranes, considering the end‐of‐cycle assessment and cost analysis of recycling of membranes. Chang et al.^[^ ^15^ ^]^ reviewed the recycling of end‐of‐life polymeric membranes. They have critically analyzed the comparative life cycle assessment and techno‐economic analysis. Tian et al.^[^ ^16^ ^]^ followed a similar approach in their review paper and reported the recycling of polymeric membranes for water treatment. Khanzada et al.^[^ ^17^ ^]^ reviewed the sustainability of membrane recycling and fabrication using recycled waste. However, these review papers could not discuss the challenges and limitations of each potential regeneration process. In addition, the impact of AI models on the regeneration of polymer‐based membranes for desalination has not been analyzed in the reported literature.

This paper aims to explore the synergistic relationship between AI, membrane regeneration, and end‐of‐life membrane management, elucidating the principles, challenges, opportunities, and emerging trends. By examining the role of AI models in monitoring, and controlling of regeneration processes, this paper provides deep insights into the transformative potential of AI in reshaping the landscape of membrane regeneration. Limitations, significant gaps, new opportunities and prospectives on the regeneration of membranes using AI are critically analyzed in this paper.

## 2 Membrane Cleaning and Regeneration

Over time, membranes used in desalination and wastewater treatment become fouled and degraded due to the accumulation of particles, organic matter, scaling and biofilm formation, and thus, performance declined with time.^[^ ^18^ ^]^ Therefore, salt passage increases through the membrane selective layer and the rejection rate drops. Membrane permeation flux also drops due to blockage of the selective layer.^[^ ^18^, ^19^, ^20^ ^]^ Understanding the types of fouling and their underlying mechanisms is crucial for developing effective regeneration strategies.^[^ ^21^, ^22^, ^23^ ^]^

**Figure 2:**
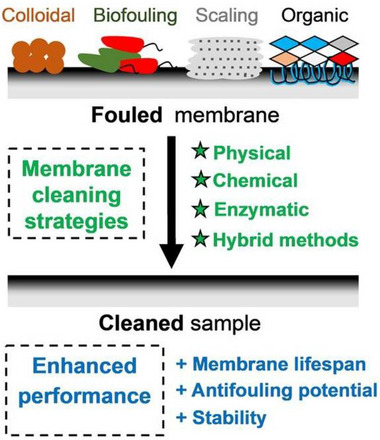
Conventional cleaning methods adopted for regeneration of membranes, reproduced with permission from ref. [26].

Inorganic fouling occurs when dissolved inorganic salts and minerals in the feed water precipitate onto the membrane surface or within its pores due to concentration polarization or changes in pH and temperature.^[^ ^24^ ^]^ Organic fouling occurs when organic compounds such as proteins, oils, polysaccharides, or humic substances adsorb onto the membrane surface or penetrate into pores, forming a fouling layer. Algal blooms from seawater are another issue which can produce organic fouling on membrane surface during the desalination process.^[^ ^25^ ^]^ Therefore, various regeneration methods are employed to restore the functionality of membranes.^[^ ^19^ ^]^ These methods include chemical cleaning, physical cleaning, biological regeneration, and thermal regeneration, as shown in **Figure** **2**.^[^ ^26^ ^]^ A detailed description of each process is provided below.

### 2.1 Chemical Cleaning

**Figure 3:**
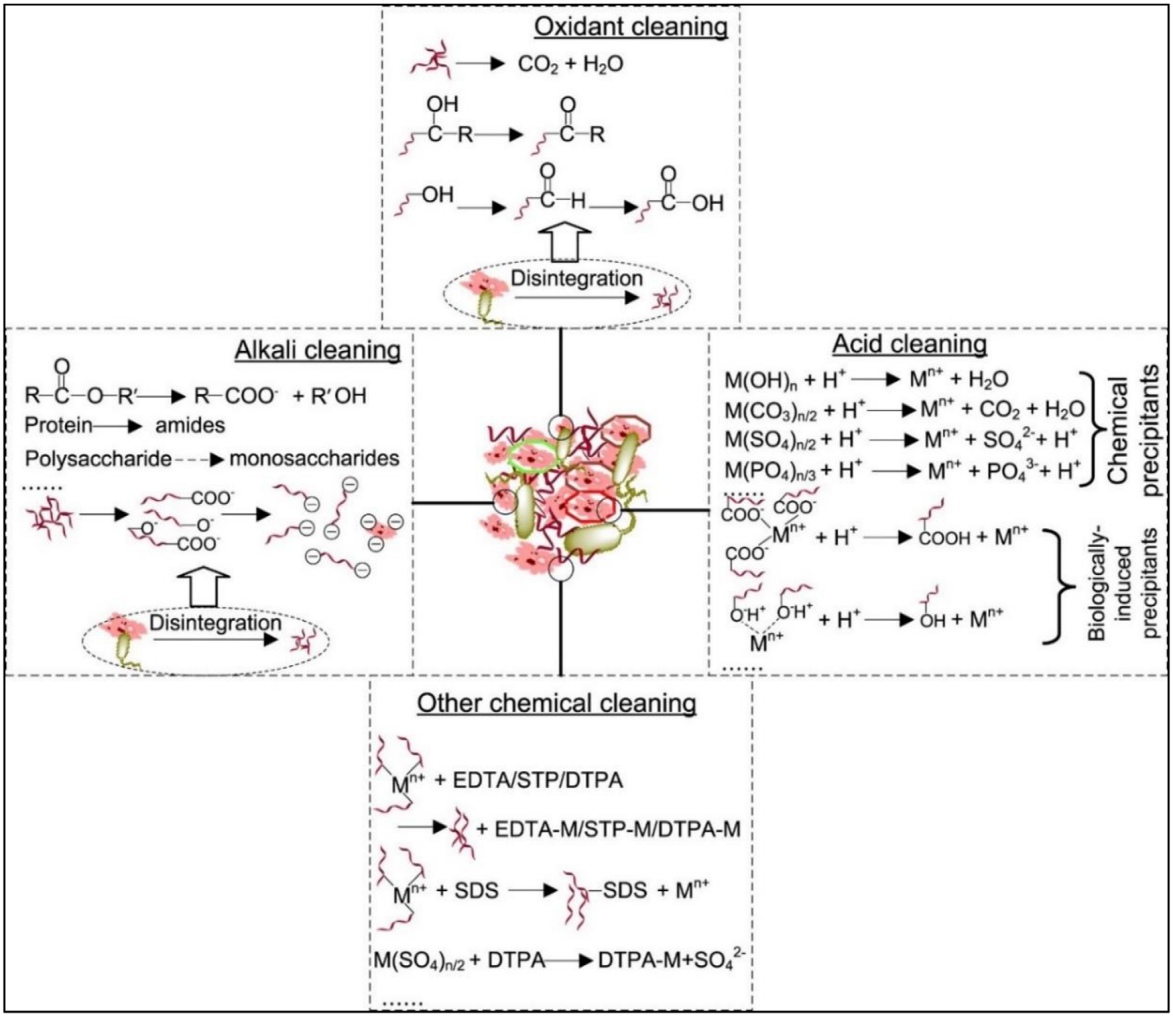
Chemical cleaning methods adopted for polymer membrane regeneration, reproduced with permission from ref. [13].

Chemical cleaning is one of the most employed methods for regenerating membranes, particularly in applications where organic and inorganic contaminants primarily cause fouling.^[^ ^21^ ^]^ Chemical cleaning can be classified into acid cleaning, oxidant cleaning, alkali cleaning, and other chemical cleaning, as shown in **Figure** **3**.^[^ ^13^ ^]^

Acid solutions such as citric acid, hydrochloric acid, and phosphoric acid are utilized to dissolve mineral deposits, scale, and metal oxides that accumulate on the membrane surface.^[^ ^13^ ^]^ Acid cleaning is effective against scaling caused by calcium carbonate (CaCO_3_), calcium sulphate (CaSO_4_), and silica.^[^ ^13^ ^]^ Sulfuric acid and hydrochloric acid are most commonly used for polyvinylidene fluoride (PVDF), polysulfone (PSF), polyamides (PA), cellulose acetate (CA), and polytetrafluoroethylene (PTFE) membranes.^[^ ^16^, ^17^ ^]^ However, these acids can damage the surface of membranes which reduced the mechanical strength after treatment.^[^ ^27^ ^]^ Besides, acid cleaning is not effective for the removal of CaSO_4_ and silica scaling.^[^ ^13^ ^]^ Therefore, alkali solutions including, sodium hydroxide (NaOH), potassium hydroxide (KOH) or other alkaline solutions are used to clean membranes because of their ability to dissolve organic foulants, grease, and biological matter from a number of distinct types of membranes.^[^ ^22^, ^28^ ^]^ Surfactants and detergents are used to mobilize and emulsify organic compounds which enabling them to be easily released from the membrane interface.^[^ ^29^ ^]^ Surfactant cleaning is effective for oils, fats, proteins, surfactants present fouling, etc.^[^ ^29^ ^]^ However, they fail to desorb the metal ions and metal oxides from the surface of membranes. Hence, chelating agents like ethylenediaminetetraacetic acid (EDTA) and citric acid are used to complex metal ions and solubilize metal oxide deposited on the membrane surface.^[^ ^30^ ^]^ Recent research indicates that microbial biosurfactants like rhamnolipids and sophorolipids can be used effectively as membrane cleaning agents because of their ability and efficiency in eliminating the fouling of organic origin.^[^ ^31^ ^]^ E.g., rhamnolipids produced by Pseudomonas aeruginosa have up to a 90% removal rate of organic fouling on the membrane. Chelating surfactants have also improved the removal of metal ion fouling and it achieved the efficiencies of up to 85%.^[^ ^31^ ^]^

Overall, chemical cleaning methods face challenges, including the multiple treatments requirement to remove all fouling materials and the unavoidable degradation of the membrane material which leads to reduce water flux and lifespan of the membrane. In addition, organic and biological fouling may not be effectively removed by chemical cleaning alone.^[^ ^21^ ^]^ This can result in incomplete regeneration and continued loss of membrane efficiency. Many chemical cleaning agents used in membrane regeneration are environmentally hazardous and can contribute to pollution.^[^ ^18^ ^]^ This limits the range of cleaning options available for certain membrane systems.

### 2.2 Backwashing

Backwashing is a hydraulic method for cleaning, and it is commonly applied in pressure‐based membranes such as reverse osmosis (RO), nanofiltration (NF), ultrafiltration (UF), and microfiltration (MF).^[^ ^28^ ^]^ It is the process of utilizing feed water or a cleaning solution to flow in the opposite direction through the membrane module to remove a foulant layer and particles, such as colloids. In hydraulic backwashing, the senses of filtration are changed by increasing pressure in the permeate side or by the extra pump, well known as the backwash pump.^[^ ^32^ ^]^ Since, water flowrate is high and thus, foulants are removed and transported away from the surface of the membrane, eliminating concentration polarization and reducing fouling, and consequently improving permeability and flux.^[^ ^32^ ^]^ Both air or gas scouring and crossflow filtration are the two types of this method. Amongst all, air scouring is most effective in preventing cake layer formation and colloidal fouling.^[^ ^33^ ^]^

Crossflow filtration and tangential flow filtration are as the name suggests, the process where the feed water directly flows parallel to the membrane surface.^[^ ^34^ ^]^ This flow configuration reduces the settled foulants on the surface of the membrane by periodically removing particles and colloids. Crossflow filtration does away with frequent cleaning of membranes and increases their useful life. This method has been applied in the regeneration of polyimide (PI)‐based membranes.^[^ ^33^ ^]^ Nevertheless, this process utilizes a large amount of water, chemicals, and other solvents for washing membranes.^[^ ^35^ ^]^

Overall, backwashing is a method in which pressure‐based membranes like RO, NF, UF, and MF air scouring, often combined with hydraulic backwashing which enhances the cleaning by using compressed air and inert gas to remove foulants. Crossflow filtration reduces fouling, but it requires significant water and chemical usage. Compared to all methods, air scouring is highly efficient and low in cost. Therefore, it is adopted at commercial desalination plants.

### 2.3 Thermal Regeneration

Thermal regeneration is utilized to remove organic foulants, biofilms, and certain inorganic deposits from membrane surfaces by applying elevated temperatures. Thermal regeneration can be applied in various forms, including hot water cleaning, steam cleaning, and chemical‐enhanced thermal regeneration.^[^ ^36^ ^]^ Hot water cleaning involves circulating heated water through the membrane module to dissolve and remove organic foulants and biofilms. The temperature and duration of hot water cleaning depend on the membrane material and the extent of fouling.^[^ ^37^ ^]^ Hot water cleaning is effective against protein fouling, microbial contamination, and organic deposits.^[^ ^38^ ^]^ Usually, temperature of hot water is kept in range of 35–40 °C. How steam cleaning process utilizes high‐temperature steam to sterilize membranes and remove organic contaminants, bacteria, and biofilms, this method has been used to remove biofilms from polyimide‐based membranes. However, steam cleaning is not suitable for most polymeric membrane materials due to the risk of thermal degradation.^[^ ^38^ ^]^ In addition, this method is limited to the short duration of cleaning.

Chemical‐enhanced thermal regeneration combines heat treatment with chemical cleaning agents to enhance membrane fouling removal efficiency.^[^ ^28^ ^]^ The synergistic effects of high temperature and chemical reactivity facilitate the breakdown and dissolution of foulants and biofilms. This method minimizes the need for aggressive chemicals and prolongs membrane lifespan.^[^ ^28^ ^]^ This method has been successfully used for the regeneration of membranes at industrial‐scale applications.

Overall, thermal regeneration method is energy intensive and requires a significant amount of energy, which limits its application at commercial scale.^[^ ^39^ ^]^ In addition, this method is not applicable to polymer membranes due to the degradation process which lead to compromise the membrane integrity.

### 2.4 Biological Regeneration

Biological regeneration is a process that employs the metabolic activity of micro‐organism and enzyme in order to remove foulants and biofilms that usually deposit on polymer membrane surfaces.^[^ ^40^ ^]^ This method is specifically useful for purposes of industrial wastewater treatment as well as water desalination since the membranes are prone to biofouling.^[^ ^41^ ^]^ Biological regeneration can be classified into two methods including, bioaugmentation as well as enzymatic cleaning. Due to the difference in the fouling behavior of individual foulants and the requirements of the membrane system, both methods provide benefits and are chosen appropriately.

Bioaugmentation is the process of putting aims microbial errs and auricle populations to the membrane systems. These microorganisms are selected for their aptitude to break down specific organic pollutants and biofilms which are associated with fouling.^[^ ^42^ ^]^ In this way, bioaugmentation guarantees that the deposited microbes compete with the other communities for resources and places to attach themselves.^[^ ^42^ ^]^ This competitive behavior keeps the biofilms to the lowest level possible and minimizes the accumulation of organic fouling on the membranes.^[^ ^43^ ^]^ Bioaugmentation is effective when the introduced microbial species can survive in the membrane environment and effectively degrade certain organic substances.^[^ ^43^ ^]^ This method is important if a native microbial community cannot effectively degrade the fouling materials. Bioaugmentation facilitates microbial degradation, the concepts help improve membranes’ performance, increase membrane durability and reduce the frequency of chemical cleaning, thus making the operations more sustainable.^[^ ^43^ ^]^

Another biological regeneration method is enzymatic cleaning in which enzymes are used to digest the foulants. Proteases and lipases are enzymes remembered as biological catalyst that implements the degradation of proteins and lipids adhering to the membrane surfaces.^[^ ^44^, ^45^ ^]^ Enzymatic cleaners are effective in breaking down these organic molecules into simpler soluble elements that can be washed away from the membrane. Compared to all methods, this method is quite selective in achieving its goal of removing foulants without compromising the membrane material.^[^ ^44^, ^45^ ^]^ In enzymatic cleaners, the use of chemical substances is lower than chemical cleaners. Therefore formation of second pollutants in this method is limited.^[^ ^33^ ^]^ This advantage makes enzymatic cleaning a safer and environmentally friendly method as compared to other methods. Enzymatic cleaners are used in regular cleaning operations or a single application to effectively remedy a particular problem with fouling. Usually, this method is widely reported for the regeneration of CA and PSF membranes.^[^ ^44^, ^45^ ^]^ However, it is costly which makes it limited to membranes principally fouled by biofilms only.^[^ ^44^ ^]^

### 2.5 Electrochemical Regeneration

**Figure 4:**
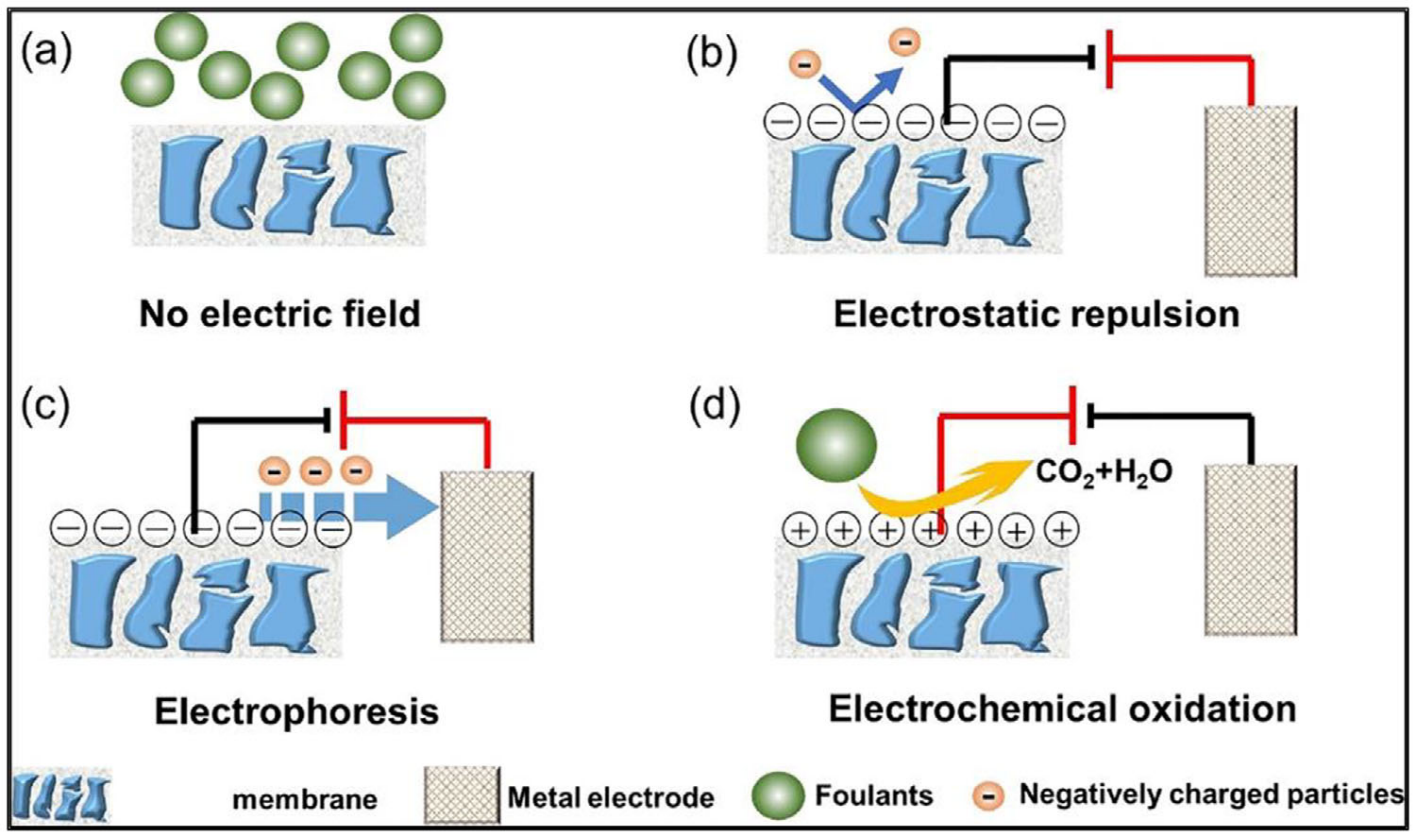
Electrochemical membrane regeneration, reproduced with permission from Ref. [22].

Electrochemical regeneration has become one of the most promising method to restore fouled membranes through an application of direct electric current as shown in **Figure** **4**.^[^ ^22^ ^]^ This method uses electric field for the prevention of membrane fouling where the membrane is regenerated by cleaning its surface. Electrochemical approach can change the properties of foulants which impacts on the surface of the membrane and thus, it induces electrochemical reactions to remove the deposits of fouling.^[^ ^22^ ^]^ During electrochemical regeneration, oxidizing species like hydroxyl radicals, chlorine, and its derivatives are produced at the anode surface by the reduction of water and halide ions. These reactive oxidants engage organic foulants and biofilms on the membrane surface to oxidize, degrade, and remove them. Consequently, the membrane surface is returned to a clean and operational state and thus, performance of membrane is enhanced.^[^ ^22^ ^]^

Another advantage of electrochemical cleaning is that it does not require any chemicals for cleaning purposes. This approach is most suitable for reversible fouling, such as dissolved foulants, including dissolved organic matter and colloidal suspension.^[^ ^22^ ^]^ Electrochemical regeneration is flexible and it can be implemented on all types of membranes including, polymeric, mixed matrix, composite and ceramic types of membranes. Furthermore, this method guarantees that the membrane material is intact and does not undergo failure during the cleaning process. In general, electrochemical regeneration is rather effective in keeping membrane surfaces clean and preserving membrane integrity when chemical application is to be limited.^[^ ^22^ ^]^

### 2.6 Summary and Prospectives on Membrane Regeneration

In summary, membranes applied to desalination faces various challenges, such as fouling, degradation, and scaling, which negatively impact the efficiency of the membrane.^[^ ^2^ ^]^ These challenges contribute to high operational costs, reduced efficiency, and pollution through constant replacement and chemical tendering of membranes.^[^ ^28^ ^]^ As with crystallization, scaling also leads to the deposition of mineral salts, which would worsen the membrane's performance and durability. In this regard, several regeneration methods are applied to rejuvenate the membranes’ performance and their useful life expectation. Techniques of regeneration comprise of chemical cleaning, backwashing, thermal regeneration, biological regeneration and electrochemical treatments. All these methods have their own positive attributes and disadvantages.

E.g., chemical cleaning was efficient in controlling the organic foulants, while its efficiency in controlling mineral deposition is low. On the other hand, biological regeneration involves microbial processes that reduce fouling substances, while this method required longer duration than the chemical method for fouling materials.^[^ ^22^ ^]^ An important consequence of the above diversity is the possibility of developing regeneration solutions that are optimized with reference to the all types of membranes used and the peculiarities of the operational conditions of desalination processes.^[^ ^22^ ^]^ Compared to chemical cleaning approach, backwashing method emerges as the simplest and most used at a large scale and with the lowest cost. It entails operating the system in the opposite direction of the foulant transport to wipe off foulants from the surface of the membrane, which makes it a viable process for those systems that required frequent regeneration.^[^ ^22^ ^]^ Compared with other complex approach, backwashing has the advantages of simplicity, low operating cost, and easy retrofitting. Therefore, it is favored by many large‐scale systems. In the meantime, electrochemical cleaning has risen as lead method for renewing RO and NF membranes. This method is highly recommended due to its low energy functionality, which may range from 0.1 to 2 kWh to restore membrane function.

In conclusion, all methods of membrane regeneration seem to be effective; however, the choice of the method should be made depending on the type of fouling, the type of membrane material and operational conditions. The application of regeneration methods remains relevant in advancing the sustainability and operational effectiveness of desalination, demonstrating the value of innovative and versatile approaches.

## 3 Challenges and Issues with Regeneration of Membranes

The regeneration of membranes is challenging as multiple issues directly impact membrane integrity during the regeneration process. **Table** **1** shows the challenges associated with membrane regeneration. A detailed description of each issue is given below.

**Table 1:** Challenges and issues with the regeneration of membrane.

Challenge with membrane regeneration	Description	Method to minimize the regeneration challenges	Advantages of regeneration method	Challenge with regeneration method	Type of membrane regeneration	Refs.
Chemical degradation & membrane integrity	Accumulation of organic, and biological materials on the membrane surface, reducing efficiency and lead to block the pores	Chemical cleaning, Physical cleaning such as chlorine disinfection Backwashing	Restores membrane performance, extends lifespan, reduces operational downtime	Can be costly, potential for chemical damage to membrane. May not remove all fouling types	Polyvinylidene fluoride (PVDF), polysulfide (PS), polyamides (PA)	[13, 40, 41]
	Deposition of minerals like calcium carbonate and magnesium sulphate, leading to blockage and reduced performance.	Acid cleaning anti‐scaling dosing	Effectively removes mineral deposits, prevents scale formation, maintains high water flux	Acid can damage membrane, anti‐scalants may not be effective for all types of scaling	Polyamide (PA)	[13, 40]
	Exposure to harsh cleaning chemicals can deteriorate membrane material over time, reducing its effectiveness	Use of milder cleaning agents Thermal regeneration	Minimizes damage to membrane material, prolongs membrane life, maintains membrane integrity	Milder agents may be less effective, increased cleaning frequency may be required	polyvinylidene fluoride (PVDF) and polyethylene terephthalate (PET).	[27, 46, 47]
Membrane Aging	Over time, even with regeneration, membranes may not fully recover their original performance, leading to decrease the life span	Application of green solvents method can be used to minimize the membrane aging effect during the regeneration process	Reduces risk of physical damage, maintains structural integrity, allows for repeated use	Membrane integrity cannot be recovered due to aging of polymer layers	polyimide (PI) and polysulfide (PSF).	[48, 49]
Membrane Performance	Membrane permeability or water flux on permeate side	Acid cleaning Alkali cleaning	High flux of regenerated membrane can be achieved.	Acid can damage membrane surface and thus reduced the permeate flux of regenerated membrane	Polyamide (PA)	[21, 36]
System Design & Control	Process control and design to regenerate the membrane to optimize the regeneration process	AI, sensors, and automatic tools used in the regeneration process.	Online regeneration without breaking module. Lower cost of regeneration.	Involvement of AI and AI model is challenging.	Polyamide (PA)	[48, 50]
Lab scale to industry scale‐up	The regeneration process which can be used at large scale and industrial scale.	Integration of engineering and design for regeneration process	Large number of membranes. can be regenerated.	Scale‐up of regeneration process is complicated. Requires process parameters optimization	CA membrane	[29, 51]

### 3.1 Chemical Degradation and Loss of Membrane Integrity

Based on Table 1, chemical degradation and integrity of membranes is one of the major challenging issues which occurred during the regeneration of membrane.^[^ ^52^ ^]^ Polymeric membranes are susceptible to chemical degradation when exposed to aggressive chemicals, high temperatures, and extreme pH conditions.^[^ ^46^ ^]^ Understanding the chemical compatibility of membrane materials and the mechanism of degradation is essential for minimizing degradation induced performance loss and extending membrane lifespan.^[^ ^46^ ^]^ Oxidizing agents such as chlorine, ozone, and hydrogen peroxide can react with polymer chains which lead to chain scission, cross‐linking and oxidation of functional groups.^[^ ^25^ ^]^ This can lead to degradation of mechanical strength, modification of the surface characters and decrease rejection.^[^ ^47^ ^]^ Membranes damaged by oxidative degradation can be treated with reducing agents and antioxidants such as sodium borohydride, ascorbic acid, glutathione and thiourea for the restoration of membrane structure.^[^ ^25^ ^]^

Hydrolytic degradation is another problem in which membranes encounter during the process of regeneration.^[^ ^53^ ^]^ Hydrolytic degradation is a process in which polymer chain takes place in the presence of water under strongly acidic and alkaline conditions.^[^ ^53^ ^]^ This digital degradation mechanism is more appropriate for hydrophilic polymers like PA and cellulose‐based membranes.^[^ ^54^ ^]^ Renewal of hydrolytically deteriorated membranes requires neutralization of residual acidic and alkaline contaminants of the membranes to minimize further moisture content and hydrolysis.^[^ ^55^, ^56^ ^]^

Thermal degradation is another challenge which appears to take place during the regeneration process of the membranes. This degradation is a very dangerous sign for the membranes themselves.^[^ ^52^ ^]^ Polymer degradation occurs through thermal breakdown, chain rupture, and loss of the volatile constituent at higher temperature.^[^ ^55^, ^56^ ^]^ This is especially problematic for thermally sensitive polymers such as PVDF and PET.^[^ ^53^ ^]^ Regenerating thermally degraded membranes could require annealing under controlled conditions to promote molecular rearrangement.^[^ ^56^ ^]^ Membrane integrity could also be impacted by annealing process as it can lose the polymer chains.^[^ ^55^ ^]^

### 3.2 Effect on Membrane Aging

**Figure 5:**
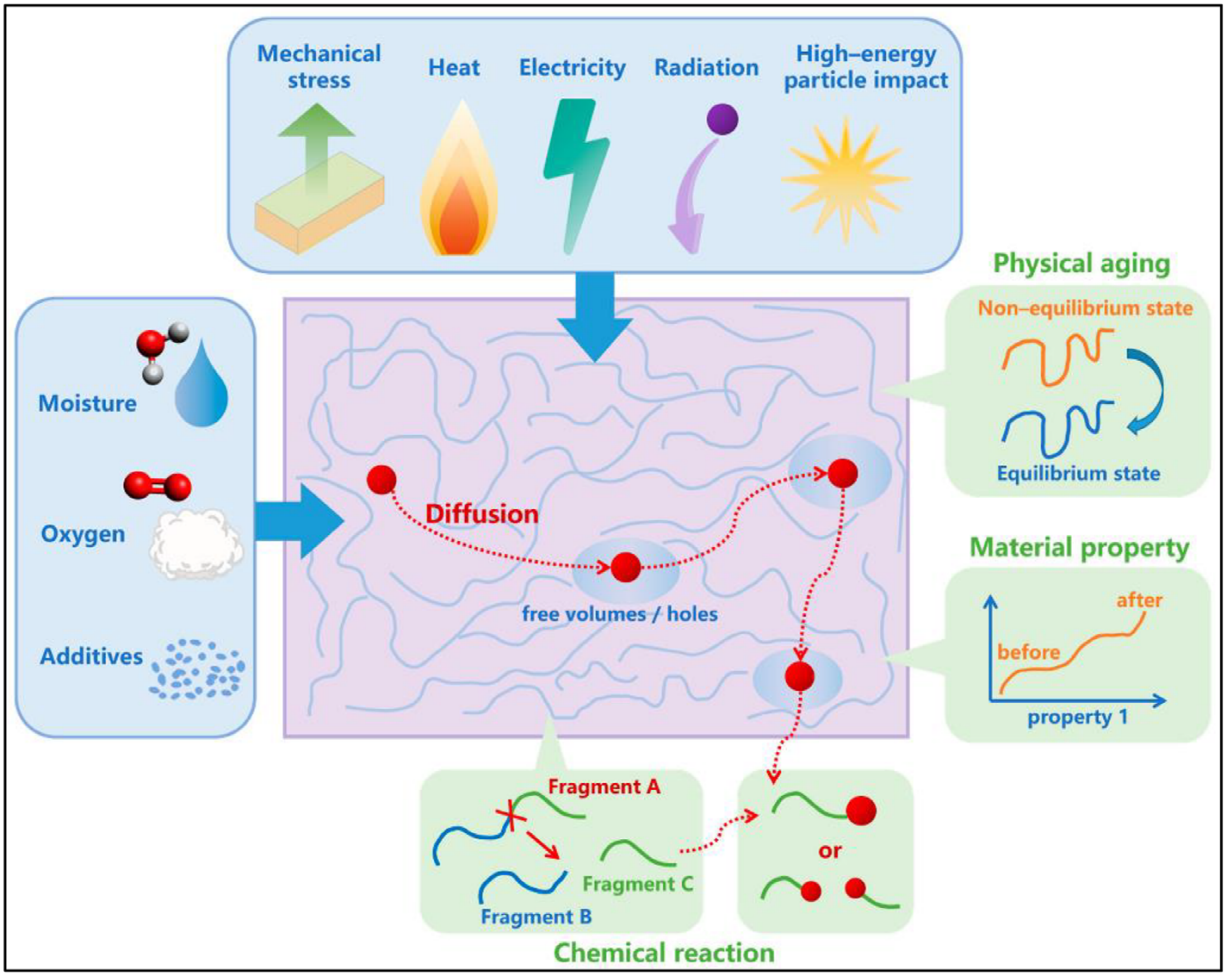
Aging mechanism in polymer membranes (reproduced with permission from paper (Zhang, F. et al.)).^[^ ^57^ ^]^

Membrane aging refers to the gradual deterioration of membrane performance over time due to various physical and chemical factors as shown in Table 1.^[^ ^48^ ^]^ Aging effects can manifest as changes in membrane morphology, surface properties, and mechanical integrity, leading to reduced permeability, selectivity, and fouling resistance. This happens to the aging mechanism as illustrated in **Figure** **5**.^[^ ^57^ ^]^ In Figure 5, parameters including, moisture, mechanical stress, heat, high energy particle impact, free volumes and pores of the polymer matrix can cause the degradation and diffusion mechanism. This results in a diffusion that activates chemical responses on the polymer matrix, breaking it into various fragments (A, B, C) and altering the properties of the material. Thermal aging is also represented showing the change of the polymer from a state of nonequilibrium to a state of equilibrium and its properties. Along with pore closing, the membrane could also face mechanical wear.^[^ ^48^ ^]^ This can increase the surface roughness, pore enlargement, and loss of membrane material, compromising separation efficiency and durability.^[^ ^58^ ^]^ Regenerating mechanically worn membranes may involve surface polishing, coating deposition, or membrane module replacement to restore smoothness and integrity.^[^ ^49^ ^]^

Usually, aging is classified as physical aging, thermal aging, and aging under mechanical stress. Thermal aging in polymer membranes is as a result of extended exposure of the membrane to high temperatures.^[^ ^58^ ^]^ This, in turn, causes step up in the rates of oxidation, chain scission and cross linking of the polymer chains. Such chemical changes can cause drastic changes in the physical characteristics of the membrane, including its flexibility, permeability, and mechanical properties.^[^ ^58^ ^]^ For instance, with thermal aging the material becomes more brittle, and the membrane is vulnerable to crack or fail.

Another type of aging is physical aging, which is time dependent structural relaxation of polymers, performed below their glass transition temperature (T_g_).^[^ ^48^ ^]^ This process results in a more tightly packed polymer structure in terms of free volume and thermostat equation. Such a densification process shrinks permeability, diffusivity, and selectivity and thus affect the functionality of separation and filtration process in membranes. While thermal aging is basically a process of chemical reactions, physical aging is a process of a change of structure of the polymer.^[^ ^49^ ^]^

For polymer membranes that are subjected to mechanical stress either in continuous or cyclic method which deforms and creeps it,^[^ ^48^ ^]^ such stress can lead to microcrack formation and localized plastic deformation causing the membrane structure to be weakened.^[^ ^48^ ^]^ Long‐term cumulative stress affects the durability and lifespan of the polymer membrane.^[^ ^49^ ^]^ Load factors that include strain rate, temperature, and the polymer composition of the membrane reduced its response to mechanical loads.^[^ ^58^ ^]^ To increase the level of resistance, membranes can be incorporated with fillers for relieving stress.^[^ ^59^ ^]^ Usually, regenerated membrane after aging also faces an issue of surface roughness which introduced the irregularities and defects on the membrane surface.^[^ ^59^ ^]^ These surface irregularities can promote fouling by providing sites for foulant attachment and entrapment. Regeneration of roughened membranes required surface modification using chemical etching, plasma activation, and coating application to smoothen the surface and improve fouling resistance.^[^ ^59^ ^]^ However, these methods have not been adopted for the regeneration of spiral wound membranes at industrial scale.

Plasticization is another issue which occurs when membrane polymers absorb solvents from the feed stream which caused the swelling, softening, and loss of mechanical strength during the regeneration.^[^ ^59^ ^]^ This phenomenon is especially prevalent in solvent‐resistant membranes such as polyimide (PI) and PSF. Regenerating plasticized membranes also involve solvent extraction, membrane conditioning, and selective barrier coatings to mitigate plasticization effects and restore membrane performance.^[^ ^59^ ^]^

### 3.3 Effect on Membrane Performance

Maintaining the flux of regenerated membranes is another challenge that needs to be addressed critically. Achieving membrane regeneration with higher efficiency requires careful selection of regeneration protocols and optimization of cleaning parameters to ensure thorough removal of foulants without causing damage to the separation properties.^[^ ^38^ ^]^ Usually, incomplete regeneration and chemical compatibility are major factors that directly impact the performance of membrane after the regeneration process.^[^ ^21^ ^]^ Incomplete cleaning of foulants and residual contaminants on the membrane surface and within its pores after regeneration can reduce the performance of the membrane due to ineffective cleaning agents, insufficient cleaning time, or incomplete rinsing steps.^[^ ^21^ ^]^ Regenerating membranes with incomplete cleaning may require alternative cleaning strategies to achieve satisfactory fouling removal and restore membrane performance. Regeneration chemicals must be compatible with membrane materials and operating conditions to avoid chemical degradation, swelling, or other adverse effects.^[^ ^45^ ^]^ Compatibility includes pH compatibility, solvent compatibility, temperature limits, and compatibility with additives.^[^ ^45^ ^]^ Regenerating membranes with incompatible regeneration chemicals necessitate the development of custom formulations and alternative regeneration methods to minimize membrane damage and ensure effective fouling removal.^[^ ^60^ ^]^

During the regeneration of membranes, maintaining selectivity and rejection rate is critical and challenging for preserving separation efficiency in membrane‐based desalination processes.^[^ ^60^ ^]^ Usually, pore size and membrane surface topology are two key parameters that are impacted during the regeneration process and, thus, require significant control.^[^ ^60^ ^]^ Regeneration processes must preserve the original pore size distribution and hydrodynamic radius cut‐off of the membrane to maintain selectivity for target solutes while removing foulants.^[^ ^61^ ^]^ However, chemicals used for the regeneration of membranes could close the pores of membranes. In addition, it can also impact on pore size as well as on the pore structure which result in a change in sieving properties, rejection mode, and separation performance.^[^ ^62^ ^]^ Therefore, pore modifiers, pore size characterization, and surface modification approaches may be needed in regenerating membranes with different pore sizes.^[^ ^63^ ^]^ For instance, if the pore size of a membrane has been changed because of fouling or degradation, then it can be reverted by using pore modifiers for example, PEG.^[^ ^64^ ^]^ Common approaches of modifying the surface of the membrane involve coating the surface, grafting and functionalization with targeted species in order to improve membrane selectivity.^[^ ^63^ ^]^ For instance, the use of hydrophilic polymer coating reduced the protein fouling in UF membranes. In the meantime, application of polymer brush layer on the membrane surface enhances membrane antifouling. Membrane surface chemistry modification is another approached which has been significantly adopted for pore modification. The functionalization with the addition of carboxyl and amine groups that would act as appropriate molecular attractors or repellents toward specific substances to favor the permeation of these substances through the membrane.^[^ ^63^ ^]^ Recently, Yuling et al.^[^ ^65^ ^]^ reported that the regenerated PA membrane had faced the issue of a lower rejection rate, and thus, it was further modified via a grafting method to enhance the rejection rate. PA membrane was grafted using different chemical agents, including, H_2_SO_4_ and NaOH for 48 h, which controlled hydrolysis of PA groups, and thus, it modulated the crosslinking degree, pore size, and surface charge of PA membrane. Therefore, separation factor of Na_2_SO_4_ was increased from 2.7 to 5.4 which is 97.1% increment while permeability improvement of water was 93.3%.^[^ ^63^ ^]^ These results indicate that the membrane functionalization has significant impact on the regeneration of membrane performance.

### 3.4 System Design and Control

System design and control parameters such as membrane geometry, flow patterns, and membrane orientation influence the efficiency and effectiveness of membrane regeneration processes.^[^ ^50^ ^]^ Therefore, it is important to design and control the regeneration process by optimization of process parameters. Usually, when membrane geometry is flat, it will be easy to regenerate the membrane surface because it can come in contact directly with a regenerating agent. Regeneration of spiral‐wound and hollow fiber membranes is challenging and requires precise injection of the regenerating agent.^[^ ^65^ ^]^ Development of modules for regenerated membranes is another challenge that leads to lower performance and added the extra cost. Therefore, the development of integrated system design is necessary to facilitate the uniform distribution of cleaning agents to improve the hydraulic performance, and to minimize the dead zones where fouling may accumulate.^[^ ^66^ ^]^ Integrated cleaning systems include online monitoring, automatic backwashing, and chemical dosing, streamline regeneration operations and improve operational flexibility. Moreover, periodic maintenance and membrane integrity testing are essential aspects of system design to ensure long‐term reliability and performance consistency.^[^ ^67^ ^]^

### 3.5 Laboratory‐Scale to Industrial scale

Currently, spiral wound and hollow fiber membranes at the industrial scale are not regenerated because of challenges including, non‐homogenous regeneration and high cost.^[^ ^38^, ^68^ ^]^ However, these membranes have been successfully regenerated at a laboratory scale. Scale‐up from laboratory to industrial scale is complicated as issues in transformation from laboratory‐scale studies to industrial‐scale increases.^[^ ^38^ ^]^

Feed water quality, fouling rate, and operating conditions also add additional challenges during scale‐up work. Therefore, process modeling and simulation tools are needed to predict the system behavior and performance.^[^ ^51^ ^]^ The main areas for consideration when scaling up are equipment design, system integration, and regulatory issues that are unique to large‐scale employment. The AI‐enabled process modeling and simulation tools provide an opportunity to model the regeneration process in a virtual environment that can help in scaling up at commercial scale industries.^[^ ^69^, ^70^ ^]^

Complex issues related to the regeneration of polymeric membranes need an interdisciplinary approach implied by cooperation between material scientists, chemists, biologists, and practitioners. By identifying the fundamental processes that control fouling, degradation, aging, and selectivity loss, it is possible to design appropriate regeneration concepts for the respective membrane material.

## 4 Role of AI in Membrane Regeneration

**Figure 6:**
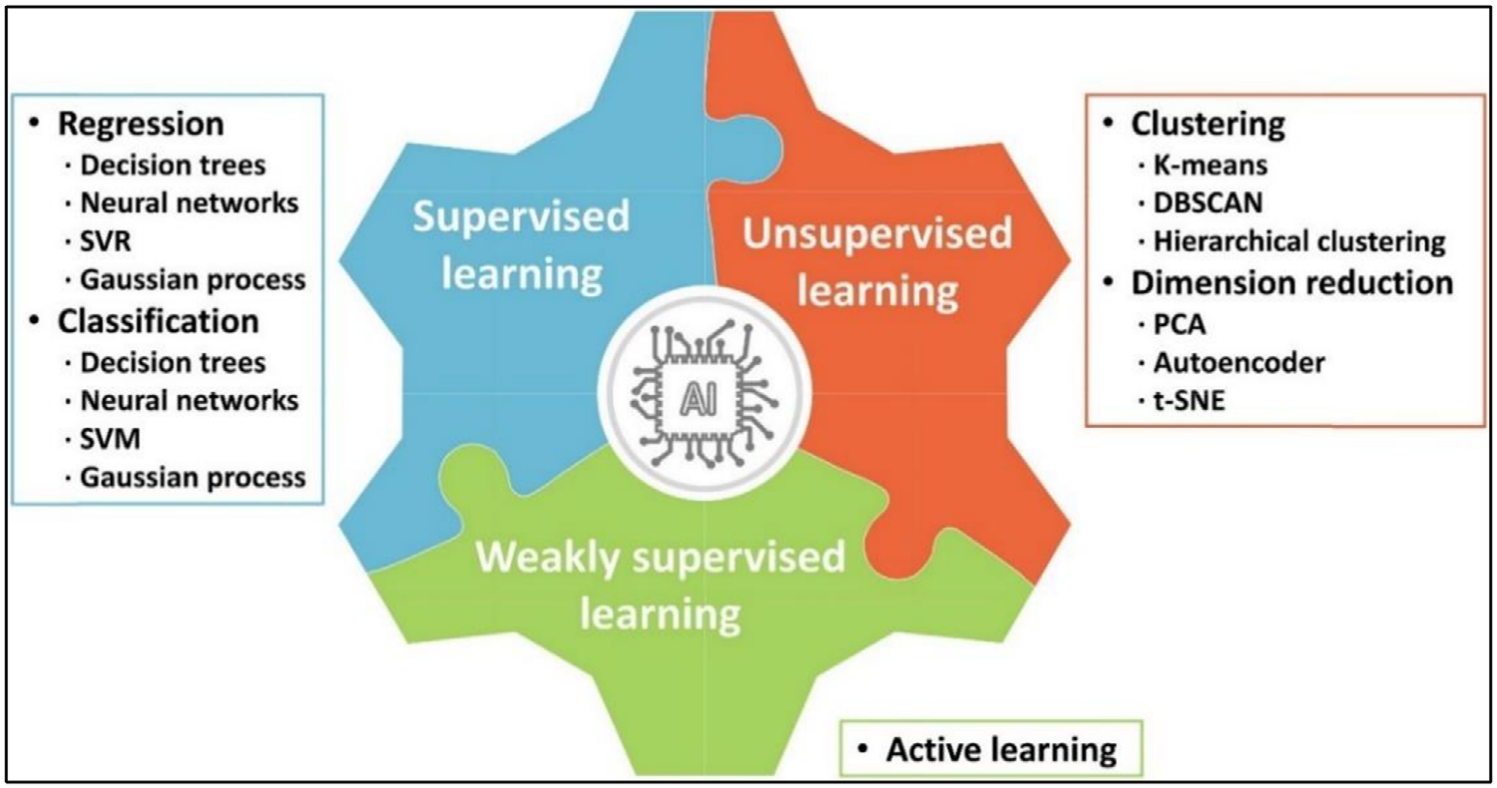
Classification of AI methods applied to membrane science, reproduced under license CC BY‐NC‐ND 4.0 from Ref. [9].

Membrane regeneration is paramount important particularly for desalination plants. Advanced technology such as the AI has improved this field through high efficiency, reliability, and sustainability in the regeneration of membranes.^[^ ^71^ ^]^ AI can be integrated to the membrane regeneration processes including process design, failure, process predictive analysis, and process optimization.^[^ ^72^ ^]^ **Figure** **6** shows the classification of AI methods which can be applied to membrane systems.^[^ ^9^ ^]^ From Figure 6, AI models are classified based on the decision trees, neural networks (NNs), support vector machines (SVMs), and Gaussian processes.^[^ ^9^ ^]^ **Table** **2** demonstrates the challenges, limitation, and role of AI models in the membrane regeneration processes including regeneration design, optimization, and failure detection.

**Table 2:** Challenges, limitation and role of AI models in the membrane regeneration.

Role in regeneration	AI model	Variable control	Power consumption	Advantages of AI model	Challenges and limitation	Refs.
Regeneration process design	GANs	Design parameters. Material properties.	7–7.2 kWh	Can generate realistic. Diverse design options. Improves design efficiency.	Requires large datasets. Computationally expensive. Requires polymer principles	[73, 74, 75]
	NNs	Process variables, Operating conditions.	0.1–0.5 kWh	High accuracy in Modeling Complex relationships.	Overfitting, Extensive training data. Extensive membrane data	[73, 74]
	SVMs	Feature selection, kernel choice.	0.1–1 kWh	Effective in high‐dimensional spaces. Robust.	Computationally intensive for large datasets. Extensive membrane data	[64]
	RF	Number of trees Depth of trees.	0.1–0.5 kWh	Handles large datasets well. Reduces overfitting	Can be less interpretable than other models	[73, 74]
Predictive regeneration and failure detection	RNNs	Time‐series data, Sequence length.	1–5 kWh	Good for temporal data, Detects patterns over time	Vanishing gradient problem, requires large datasets.	[76]
	LSTM networks	Sequence length, Memory parameters.	3–10 kWh	Overcomes vanishing gradient, Excellent for long‐term dependencies.	Complex architecture Computationally expensive	[76, 77]
Process Optimization	Q‐learning and DQNs	Learning rate, Discount factor	0.5–2 kWh for Q learning 5–15 kWh DQNs	Can optimize complex processes, Adaptive learning.	Requires significant computational resources	[78]
	Bayesian Optimization	Hyperparameters, Prior distributions.	1–5 kWh	Efficient hyperparameter tuning, Incorporates uncertainty.	Can be slow with high‐dimensional problems.	[79, 80]
	GAs	Population size, Mutation rate.	1–5 kWh	Efficient for optimization Flexible. Easy to apply on different processes. More accurate on regeneration processes.	Requires many evaluations. Multiple steps are involved.	[80]
Enhancing Filtration Efficiency	Adaptive control algorithms	Control parameters. Feedback mechanisms.	7–7.2 kWh	Real‐time adjustments, More accurate.	Complexity in design, Requires precise models. Extensive membrane data points	[81]
	Fuzzy logic systems	Temperature Pressure Composition etc., Rules sets	0.1–0.5 kWh	Handles uncertainty, Intuitive.	Rule explosion, Less precise than other methods.	[82, 83]
	Multi‐agent systems	Agent behaviors, Communication protocols.	0.1–0.5 kWh	Scalable, Distributed control.	Coordination complexity, Requires robust communication	[84]
Quality Control and Inspection	Computer vision	Detection thresholds Feature selection.	‐	Identifies defects early. Reduces downtime	Requires high‐quality images, Sensitive to lighting conditions.	[85]
	Anomaly detection algorithms	Detection thresholds	‐	Identifies defects early. Reduces downtime	False positives, Requires labeled data	[86]
	Robotic process automation (RPA)	Process workflows. Automation scripts.	‐	Requires automation	Requires automation. Still early stage Required multiple steps.	[86, 87]

### 4.1 Process Design

**Figure 7:**
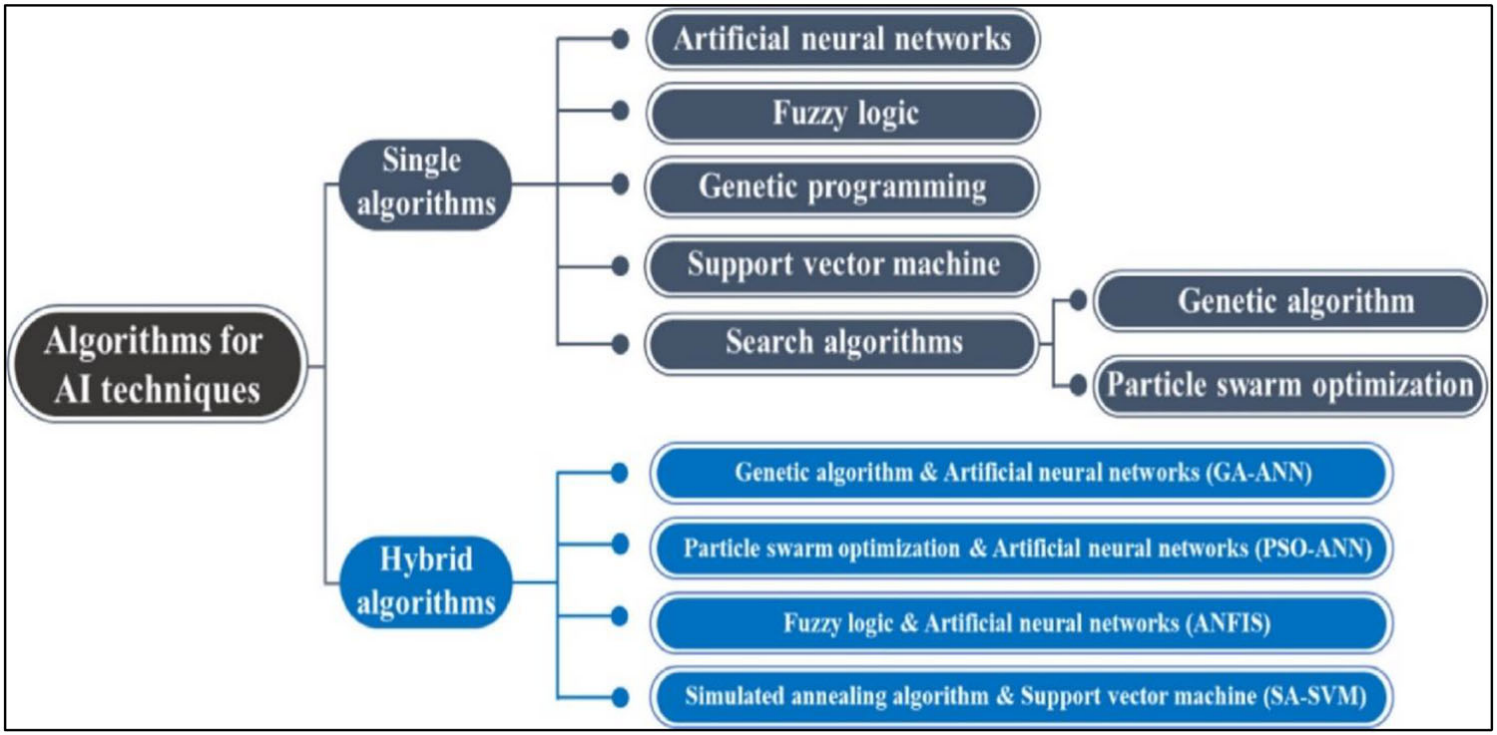
Different AI models for the membrane science and regeneration, reproduced with the permission from Ref. [76].

As shown in Table 2, AI algorithms, particularly machine learning (ML), play a crucial role in optimizing the design of the membrane regeneration process. By analyzing extensive datasets, AI can identify patterns and suggest the removal of fouling and other scaling from the membrane surface to improve the membrane properties.^[^ ^88^ ^]^ AI Models including, generative adversarial networks (GANs), NNs, SVMs, and random forests (RF) could be considered for process optimization for membrane regeneration as shown in **Figure** **7**.^[^ ^76^ ^]^

GANs models are employed to predict the regeneration process of membranes by training on existing datasets of membrane properties, including thermal stability and chemical resistance.^[^ ^75^ ^]^ The generative network in GAN models creates new designs by analyzing over 10 000 data points from various types of membranes, while the discriminative network evaluates their feasibility and performance based on specific criteria such as permeability, selectivity, and mechanical strength.^[^ ^78^ ^]^ This iterative process involves thousands of training cycles which leads to the development of membranes with improved regenerative capabilities.^[^ ^75^ ^]^ However, this model requires large datasets, often exceeding several gigabytes in size. Therefore, it is computationally expensive, with processing times ranging from several hours to days depending on the dataset size and complexity. This model also shows overfitting of the data during the regeneration of membrane‐based processes, particularly when the training data is not sufficiently diverse.^[^ ^75^ ^]^ This overfitting can lead to inaccuracies in predicting membrane performance. This model has been used for CA‐based membrane regeneration, where it involved polymer chemistry and a large dataset of over 50 000 entries related to the polymer's chemical structure, interaction with various chemicals, and environmental conditions to provide the signals for regeneration.^[^ ^65^ ^]^ This approach has shown promise in enhancing the lifespan of these membranes by ≈20%, based on preliminary testing results. However, this model has not been used in the online regeneration process. Along with GANs, NN models, particularly deep learning models, are adopted for to identify the complex patterns in large datasets in the membrane field.^[^ ^75^ ^]^ In membrane regeneration process design, they can predict structure and composition of a membrane which could contribute to the creation of highly efficient and durable membranes. Along with membrane design, these models have also been adopted for polymer‐based RO and NF membrane regeneration processes.^[^ ^89^ ^]^ However, these models require extensive training data and more than 10 000 membrane data points to achieve accurate results. SVMs and RF models can potentially be considered for NF and RO membrane regeneration process because these models have demonstrated the accuracy of 99.99% for the prediction of regenerated membrane performance.^[^ ^64^ ^]^ SVM models are computationally intensive, while RF models require less interpretation compared to the SVM, GAN, and ANN models. RF models are considered efficient models for regeneration process design.^[^ ^64^ ^]^

### 4.2 Predictive Regeneration and Failure Detection

**Figure 8:**
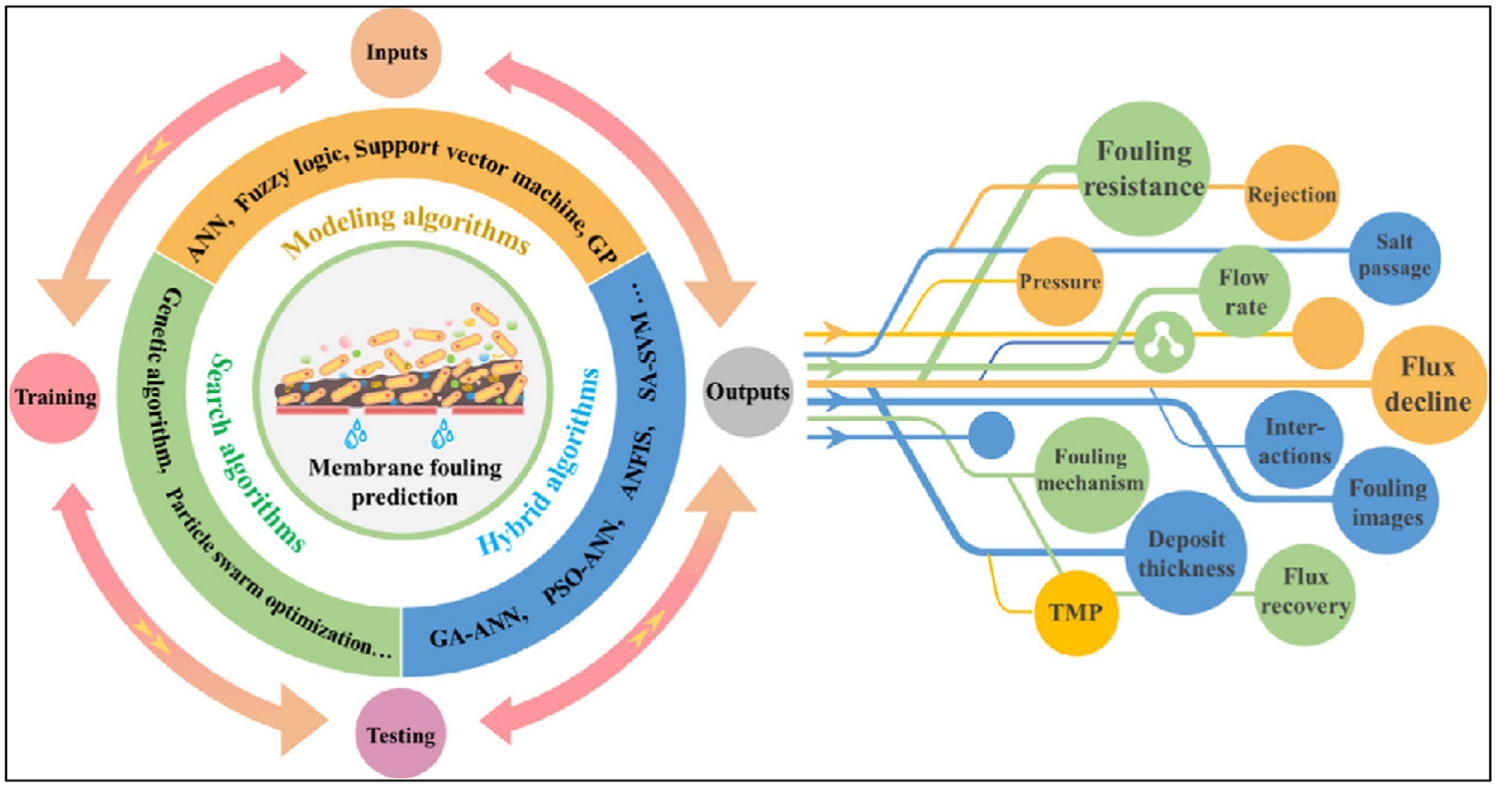
AI‐driven predictive analysis which could enable real‐time monitoring of membrane conditions, reproduced with the permission from Ref. [76].

AI‐driven predictive analytics enable real‐time monitoring of membrane conditions, predicting when a membrane is likely to fail and require regeneration.^[^ ^90^ ^]^ **Figure** **8** shows the AI‐driven predictive analysis which enables real‐time monitoring of membrane conditions. This proactive approach reduces regeneration duration and extends the lifespan of membranes. AI Models, including time series analysis models, recurrent neural networks (RNNs), and long short‐term memory (LSTM) networks, have been used to identify the polymer membrane conditions and performances in desalination processes, as shown in Table 2.^[^ ^76^ ^]^ Time series analysis models analyzed the trends and patterns in data collected over time to predict membrane performance.^[^ ^91^ ^]^ They are crucial for identifying when a membrane is likely to fail, allowing for timely maintenance and reducing operational disruptions.^[^ ^76^ ^]^ RNNs and LSTMs are particularly well‐suited for time‐series data, making them ideal for predictive maintenance and regeneration. They can learn from past operational data and predict future failures, enabling pre‐emptive action to avoid downtime and ensure continuous operation.^[^ ^77^ ^]^ Therefore, these models can be preferred over the other models for membrane regeneration processes. However, these models face the issues of complex computations which limit their application in the membrane regeneration process.^[^ ^77^ ^]^

### 4.3 Process Optimization

AI models have also been used to optimize the regeneration processes, such as cleaning protocols for fouled membranes. By analyzing the types and extents of fouling, AI can recommend the most effective cleaning strategies, reducing chemical usage, energy consumption, and improving overall efficacy.^[^ ^92^ ^]^ AI models including, reinforcement learning (RL), Bayesian optimization, and genetic algorithms (GAs) have been considered for the NF and RO membrane regeneration processes.^[^ ^78^ ^]^

RL models, such as Q‐learning and deep Q‐Networks (DQNs) possess the potential to optimize regeneration processes by continuously learning and adapting to changing conditions as shown in Table 2.^[^ ^78^ ^]^ They simulate various scenarios to find the most efficient cleaning protocols and operational settings, enhancing the effectiveness of membrane regeneration. To date, these models have been applied for the prediction of regeneration of polymeric NF, UF, and MF membranes. DQNs model demonstrated an accuracy of 99.9%. However, this model has not been applied for ceramic membranes where the inorganic and surface chemistry is different compared to polymer chains which require significant computational resources on membrane regeneration processes with datasets covering a range of variables such as temperature, pressure, and composition.^[^ ^78^ ^]^

Bayesian Optimization is another model which could be considered to optimize the process parameters including, temperature, pressure, composition, heating, and duration.^[^ ^79^ ^]^ This is a probabilistic model, and it could help to identify the optimal parameters for complex processes. In membrane regeneration, Bayesian optimization can optimize chemical usage and cleaning cycles, maximizing efficiency and minimizing costs.^[^ ^80^ ^]^

GAs models are inspired by natural selection and optimize processes by iteratively improving solutions. They identify the best combination of operational parameters to enhance membrane regeneration, ensuring efficient and sustainable operation.^[^ ^75^ ^]^ To date, these models are limited to the investigation of polymeric membrane regeneration in high fouling systems.^[^ ^75^ ^]^

Overall, Bayesian optimization could be considered as an efficient AI model for the membrane regeneration process as these models involved a large number of parameters and thus, could provide accurate results during the regeneration process.^[^ ^93^ ^]^ In addition, this model incorporates uncertainty, flexibility, and high efficiency which gives them priority over the other models, and thus, it has been adopted for process optimization over the CA^[^ ^94^ ^]^ and PA‐based membranes.^[^ ^75^ ^]^

Along with membrane regeneration process optimization, AI and ML have been used to develop adaptive filtration systems that adjust operational parameters in real time which could contribute to enhancing the filtration efficiency.^[^ ^78^ ^]^ These systems optimize process parameters such as pressure and feed flow rate passing through the membrane. Therefore, control of such parameters via AI model could also be helpful for the membrane regeneration processes as well.^[^ ^75^ ^]^ AI models such as adaptive control algorithms, fuzzy logic systems, and multi‐agent systems could be considered to use for membrane regeneration processes.

Like the other models, adaptive control algorithms adjust operational parameters in real time based on the condition of the membrane as shown in Table 2.^[^ ^81^ ^]^ They ensure that the filtration system operates at optimal efficiency by continuously monitoring and adjusting variables such as pressure and flow rate.^[^ ^95^ ^]^ Thus, applications of such a model are also used for membrane regeneration processes because these models contribute to the pore regeneration and pore design which controlled the regenerated flux of membranes.^[^ ^82^ ^]^ Like adaptive control algorithms, fuzzy logic systems handle uncertainties and approximate reasoning, making them suitable for controlling complex filtration processes.^[^ ^82^, ^83^ ^]^ They can dynamically adjust filtration parameters to maintain efficiency, which could contribute to extending polymer membrane life. Multi‐agent systems are another type of AI models in which multiple AI agents work together to manage different aspects of filtration process.^[^ ^84^, ^96^ ^]^ They coordinate actions to optimize the overall membrane regeneration process and adapt to changing conditions in real‐time, ensuring that a continuous and efficient membrane is required to be obtained.^[^ ^97^ ^]^ Decision trees are also a type of AI model that helps in understanding the relationships between membrane properties and performance. They can identify key factors that contribute to effective membrane regeneration and guide the development of new materials with enhanced regenerative capabilities.^[^ ^98^ ^]^

When all models are compared (Table 2), it has been found that the DQNs AI model is more efficient as they perform efficiently and provide more accurate results (99.9% accuracy) for regeneration of membranes. DQNs detect patterns over real time efficiently for membrane processes with higher accuracy of 99.9%.^[^ ^98^ ^]^

## 5 Limitation of AI Models in Membrane Regeneration

AI models in membrane regeneration processes exhibit significant limitations including dependency on high‐quality data set points, model interpretability, computational resources, generalization, and regulatory considerations which have lowered their applications for membrane regeneration process.^[^ ^99^ ^]^

One of the major limitations of AI models is the dependence on high‐quality and extensive datasets.^[^ ^100^ ^]^ In desalination processes, collecting comprehensive and accurate data can be challenging due to sparse sampling, systematic errors in analytical instruments and the variability of feedwater quality, permeate quality, rejected water quality, cleaning in process control and many more. Incomplete and noisy data can lead to inaccurate predictions and suboptimal decisions, which ultimately impact on the regeneration of the membrane process and, thus, could impact the integrity of membranes.^[^ ^101^ ^]^ E.g., a convolutional neural network (CNN) model trained on data with significant gaps and errors might misinterpret the condition of the polymer membrane regeneration process, and thus, it leads to incorrect recovery recommendations, which ultimately reduce the regeneration and integrity of the membrane.^[^ ^64^ ^]^ In addition, a CNN model offers small data set points which can provide errors for more than 30–50%, which can also result in the wrong recommendations for the recovery of membranes.

Another limitation of AI models is the interpretation of data, which further effect the membrane regeneration.^[^ ^102^ ^]^ This lack of interpretability makes it hard for operators to trust and understand the decision‐making of AI systems. Therefore, it affects the membrane regeneration process.^[^ ^103^ ^]^ Hence, ensuring the transparency and explainability of AI models is crucial for their widespread adoption in the membrane regeneration process. It is important to interpret the AI model accurately and train the operators for efficient execution during the regeneration process.^[^ ^103^ ^]^

Another limitation with AI models is the availability of computational resources.^[^ ^104^ ^]^ Advanced AI models, particularly deep learning networks, require significant computational resources for training and deployment.^[^ ^104^ ^]^ Desalination plants, especially those are in remote areas, may lack the necessary infrastructure to support these computational demands. Efficient deployment of AI solutions in such settings remains a challenge.^[^ ^96^ ^]^ Training a deep learning model on‐site for membrane regeneration is impractical due to limited computational power and connectivity, necessitating off‐site processing. This will unavoidably reduce delays and thus, reduce the efficiency and increase the cost of regeneration management.^[^ ^78^ ^]^

Application of AI models into the existing membrane desalination system can be complicated, and it needs technology development such as installation of communication systems, sensors, and enhanced internet utilities.^[^ ^105^ ^]^ Integration between control systems, AI and traditional process control equipment entails the need frameworks and compatibility standards. Using new AI technology in older plants may require capital investment also as a part of retrofitting in the plant.^[^ ^105^ ^]^ For instance, an advanced AI‐based predictive regeneration system may require integration of AI with SCADA systems which could pose the technical difficulties. Meanwhile, the incorporation of new AI models to different conditions that facilitate the regeneration process presents another major issue which is attributed to the scarcity of data.^[^ ^106^ ^]^ Consequently, AI models learned from data derived from specific desalination plants under particular working conditions may not work under other desalination plants.^[^ ^64^ ^]^ Real‐time monitoring, feedwater composition, and membrane types, as well as operational practices, can hinder AI solution generalizability.^[^ ^106^ ^]^ Therefore, this is a limitation that researchers must explore, and AI models must be trained for different uses at different desalination plants.

Integration of AI in the regeneration of polymer membranes is a process that entails various steps, which come with cost implications. Hardware and software take a large proportion of the total cost of investment, while licenses for AI software required for ML and predictive analytics are in the range of $ 5000 to $ 50 000 per year.^[^ ^104^, ^105^ ^]^ Besides, other hardware resources are required which include GPUs and special servers which is generally between $ 10 000 and $ 100 000. AI software licenses and predictive analytics can cost between $ 5 000 and $ 50 000 per year.^[^ ^105^ ^]^ Additionally, high‐performance computing resources such as GPUs and specialized servers may be required, with initial hardware costs ranging from $ 10 000 to $ 100 000.^[^ ^78^ ^]^ Another cost is data collection from historical and real‐time data of membrane regeneration processes which could cost between $ 5000 and $30 000, depending on the scale of the collection. The storage and processing of these large datasets could add another $ 1000 to $ 10 000 per annum to the total expenses. However, data cleaning and labeling feeding into an AI model, might still need manual assistance; the cost estimate runs approximately between $ 10 000 to $ 50 000.^[^ ^78^ ^]^

Most of the AI models require hiring and contracting specialists and data scientists. Therefore, their wages range could be between $100 000 to $200 000 per employee annually. Moreover, integrating AI tools and systems can also come with the extra expense of $ 5000 to $ 20 000 for training the current staff for utilization of these tools.^[^ ^78^ ^]^ Investment to keep the AI system as current also plays a critical role in sustaining the use of membrane regeneration. Updating the AI program may require an expense of 20–30% of the cost of the initial purchase. Further updates and optimization of AI models might cost from $10 000 to $50 000 per year.^[^ ^78^ ^]^ Training existing staff to use AI tools and systems might incur additional costs of $ 5000 to $ 20000 per session, depending on the depth and duration of the training.^[^ ^78^ ^]^ Ongoing maintenance and updates are also necessary to ensure the long‐term success of AI in membrane regeneration. Annual maintenance and updates for AI software might cost around 20–30% of the initial purchase price. Continuous improvement of AI models could require an additional $ 10 000 to $ 50 000 annually.^[^ ^78^ ^]^ Intensive surveillance to ensure that the AI systems are running properly also requires a set of human resources and external support services which will cost an additional $10 000 per year.^[^ ^78^ ^]^

Finally, additional expenses such as regulatory costs come into the picture. The implication and compliance with regulatory requirements in the AI implementation process would necessitate a legal bill and compliance audit, which may set buyers back between $5000 and $20000 every financial year. Furthermore, there can be reduced productivity during the integration phase of the project which can induce an additional cost.^[^ ^98^ ^]^ Altogether, it costs from little to thousands of dollars to adopt the AI into the polymer membrane regeneration process.^[^ ^64^ ^]^ Therefore, the initial implementation might take approximately from $ 500000 to $ 1.0 Million or even more, depending on the context of the project.^[^ ^98^ ^]^ Meanwhile, small‐scale implementation might cost around $ 100000 to $ 200000 depending on the specific requirements and scale of the project.^[^ ^98^ ^]^

## 6 Future Directions and Prospectives

**Figure 9:**
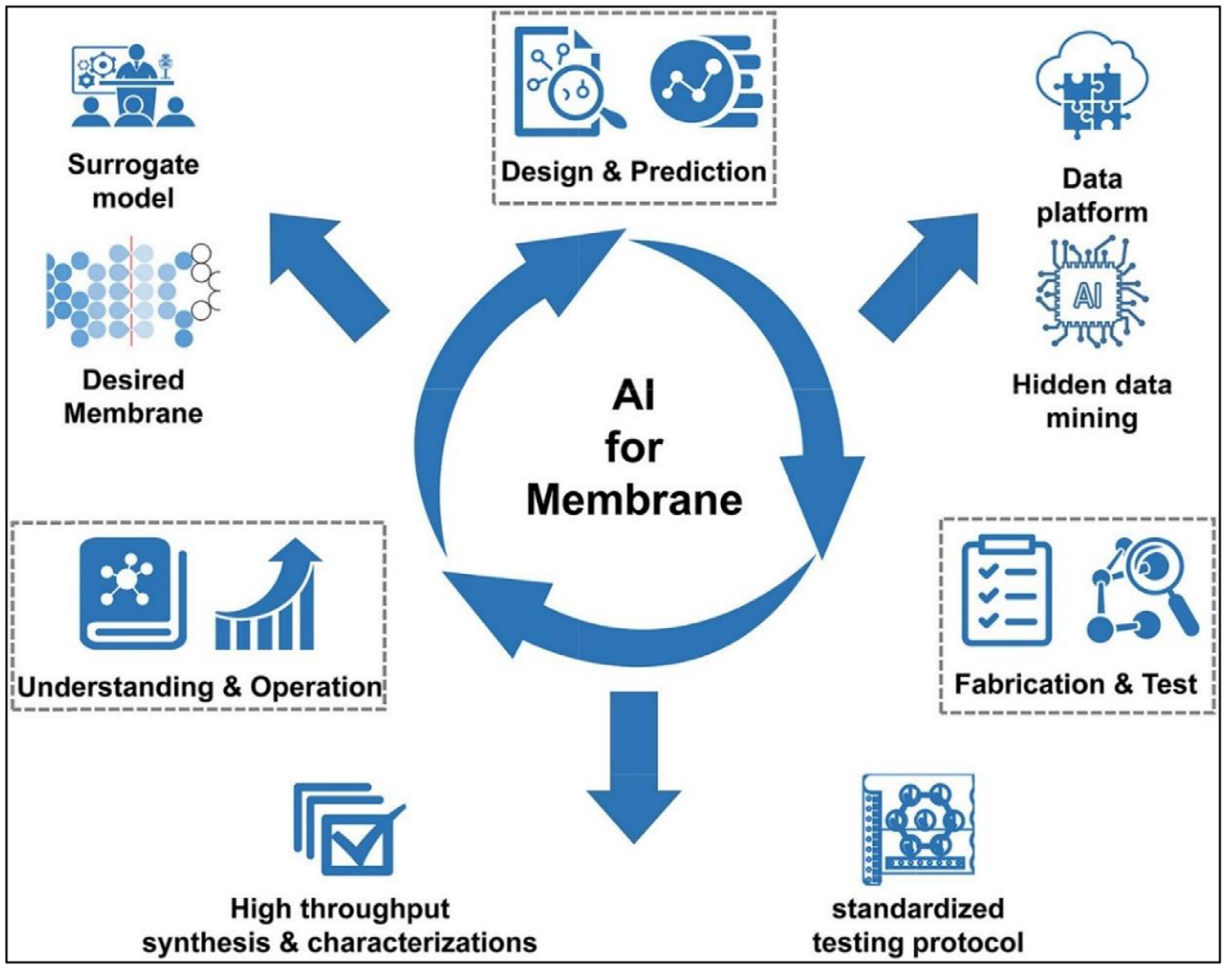
Future directions of AI for membrane regeneration and process design, reproduced under license CC BY‐NC‐ND 4.0 from Ref. [9].

Current membrane regeneration processes are conducted manually and require significant effort to make it online regeneration as it can save the cost of regeneration process. Recently, AI models such as the Bayesian model have been used for regeneration of polymer‐based membrane. AI models can optimize chemical usage and cleaning cycles, maximizing efficiency and minimizing regeneration cost, with reported accuracy of up to 99% for the regeneration process.^[^ ^107^ ^]^ Therefore, an advanced AI model‐based regeneration system needs to be developed, which could provide high‐resolution data on membrane performance and water quality to enhance the capabilities of the online regeneration systems.^[^ ^108^ ^]^ The development of sensor technology will facilitate more accurate regeneration of membranes over the desalination process.^[^ ^109^ ^]^ Sensors and AI models can be capable of real‐time and in situ measurement of thickness on membranes to provide valuable data for AI models for removal of fouling from the membrane. Thus, it may be possible to restore a membrane to its original performance with the help of regenerative systems. **Figure** **9** shows the future directions of AI for membrane regeneration and process design.^[^ ^9^ ^]^ From Figure 9, combining different AI techniques with ML, deep learning, and expert systems could create hybrid models that leverage the strengths of AI models and ML, deep learning, and expert systems.^[^ ^110^ ^]^ Hybrid models can offer higher accuracy, robustness, and interpretability which make them more effective for membrane regeneration applications.^[^ ^110^ ^]^ E.g., a hybrid model combining a NN network for image analysis and a decision tree for interpretability could be integrated to provide an accurate fouling detection, which will further give precise signals to sensors to remove the fouling from the membrane surface.

Moreover, current AI models are not connected to the IoT and, thus, provide offline solutions for the regeneration of membranes, which can further increase the regeneration duration.^[^ ^110^ ^]^ Therefore, it is recommended to integrate AI with edge computing and the IoT which can overcome the computational challenges by processing data at the site of data generation.^[^ ^111^ ^]^ This approach reduces latency, enhances real‐time decision‐making, and enables the deployment of AI solutions in resource‐constrained environments. In addition, creating collaborative platforms for data sharing among desalination plants can enhance the availability of high‐quality datasets for AI model training. Such platforms facilitate the development of robust AI solutions which are applicable across different plants and conditions. A global database of membrane performance data from various desalination plants could be developed which could be further used to train AI models. Furthermore, sustainable AI practices such as less computational power of 0.5–1 kWh could be adopted which should be able to build the energy‐efficient algorithms to minimize the environmental impact of AI technologies on membrane regeneration systems. Usually, AI models could consume the energy consumption in range of 0.5–7.2 kWh/day during the regeneration of membrane process. Sustainable AI practices align with the broader goals of environmental stewardship and resource conservation in membrane processes can be helpful to produce the efficient regeneration system.

## 7 Conclusions

Membranes used in desalination plants require regeneration to reduce energy consumption and desalination costs. The integration of AI could present a transformative potential for enhancing the regeneration of membranes by increasing the monitoring, maintenance, and optimization capabilities, AI may boost the efficiency, sustainability, and cost‐effectiveness of the membrane regeneration process. There are several difficulties that should be solved to leverage AI in membrane regeneration which includes high data quality, better comprehensibility of models, sufficient computing resources, and compatibility with the existing structures. Extension of hybrid AI system models, use of edge computing, data sharing and sustainable AI practices are imperative for future studies. Addressing these challenges, as well as utilizing the benefits of AI, the desalination industry can improve membrane regeneration processes to a greater extent. This will not only help to operate the desalination plants in the most efficient way, but also help to achieve the long‐term goal of providing clean water in a sustainable way for future generations. However, future research should be conducted to improve the sensor technologies, the creation of hybrid AI, use of edge computing, IoT and data sharing. Conversely, if these impediments are addressed and these windows of opportunities are grasped, AI can significantly contribute to provide access to clean water.

## Conflict of Interest

The authors declare no conflict of interest.
